# Operative Versus Non-operative Management of Displaced Midshaft Clavicle Fractures: A Systematic Review of the Latest Literature

**DOI:** 10.7759/cureus.88596

**Published:** 2025-07-23

**Authors:** Luke Borg, Samuel Mifsud, Martina Vella, Stephan Grech

**Affiliations:** 1 Department of Orthopaedics and Traumatology, Mater Dei Hospital Malta, Msida, MLT

**Keywords:** clavicular fractures, functional status, non-union, open reduction and internal fixation, shoulder trauma

## Abstract

Displaced midshaft clavicle fractures in adults have traditionally been managed non-operatively; however, recent literature has challenged this approach, particularly in active populations. This systematic review aims to compare functional outcomes, union rates, complication profiles, and patient satisfaction between operative and non-operative management strategies. A comprehensive search of PubMed, Embase, and the Cochrane Library was conducted in February 2025, identifying 10 eligible studies published from 2015 onwards: eight randomized controlled trials and two prospective cohort studies. Risk of bias was assessed using the Non-randomised Studies of Interventions (ROBINS-I) and Risk of Bias 2.0 (RoB2) tools. Overall, operative treatment was associated with superior early functional outcomes and more rapid radiographic union. However, long-term functional scores and patient satisfaction were comparable between groups. Surgical intervention carried a higher incidence of reoperation, primarily due to hardware irritation. These findings suggest that while operative fixation offers early clinical benefits, non-operative treatment remains a viable and effective option for many patients, particularly those with lower functional demands or contraindications to surgery. Clinical decisions should be tailored to individual patient needs, balancing short-term gains against surgical risks.

## Introduction and background

Clavicle fractures account for approximately 2.6% to 5% of all fractures sustained in the adult population, with the midshaft segment implicated in nearly 80% of cases due to its inherent anatomical susceptibility and biomechanical stress concentration [1]. Traditionally, these injuries have been managed conservatively using immobilisation devices such as slings or figure-of-eight braces. This approach has been widely accepted due to its non-invasive nature, ease of application, and relatively low incidence of complications [2]. However, over the past two decades, accumulating clinical evidence has increasingly challenged the adequacy of non-operative treatment for displaced midshaft clavicle fractures. These injuries are now recognised to be associated with higher rates of malunion, nonunion, and persistent functional impairment when treated non-surgically [3-5].

Operative management, primarily through open reduction and internal fixation (ORIF) using plate constructs, has therefore been proposed as an alternative strategy. This technique theoretically offers the advantages of anatomical realignment, enhanced mechanical stability, and earlier return to function [6]. Nevertheless, surgical intervention carries inherent risks, including infection, neurovascular injury, hardware-related irritation, and the potential need for subsequent reoperation [7-9].

At present, there remains considerable equipoise within the orthopaedic community regarding the superiority of operative versus non-operative management for this injury pattern. Numerous randomised controlled trials (RCTs) and prospective cohort studies have addressed this clinical question, but findings have been heterogeneous. While some studies highlight short-term functional advantages with surgical fixation, others report no significant differences in long-term outcomes such as union rates, shoulder function, or patient satisfaction [10-12].

The purpose of this systematic review is to critically evaluate and synthesise recent literature, specifically studies published from 2015 onwards, that directly compare operative and non-operative interventions for displaced midshaft clavicle fractures in adults. Particular emphasis is placed on comparative outcomes related to functional recovery, fracture union, complication profiles, and overall patient satisfaction. The aim is to provide clinicians with an updated, evidence-based overview to support clinical decision-making and shared treatment planning.

## Review

Methods

PICO (Patient/Problem, Intervention, Comparison, and Outcome) Framework

This systematic review was guided by the PICO framework to formulate the clinical question. The population (P) comprised adult patients aged 18 years and older with displaced midshaft clavicle fractures. The intervention (I) assessed was operative treatment through ORIF using plate constructs. The comparator (C) included non-operative management strategies such as slings or figure-of-eight bandages. The primary outcomes (O) evaluated were functional results measured using validated scoring systems (e.g., Disabilities of the Arm, Shoulder and Hand (DASH) and Constant scores), radiographic union rates, complication rates, and patient-reported satisfaction.

This review was not prospectively registered with the PROSPERO database or any other international registry, a methodological limitation that is hereby acknowledged.

Eligibility Criteria

Inclusion criteria were as follows: studies published in English from 2015 onwards; studies involving adult participants (aged 18 years or older) with confirmed displaced midshaft clavicle fractures; investigations that provided a direct comparison between operative treatment (specifically plate fixation) and non-operative approaches; and studies that reported at least one of the following outcomes: functional scores, radiographic union, complication rates, or patient satisfaction. Only RCTs and prospective cohort studies were included.

Exclusion criteria encompassed case reports, conference abstracts, narrative or systematic reviews, studies focusing on paediatric, distal, or pathological clavicle fractures, and articles for which full-text access was not available despite reasonable efforts to obtain them.

Search Strategy and Study Selection

A systematic search of PubMed, Embase, and the Cochrane Library was conducted in February 2025. The search strategy aimed to identify studies comparing operative and non-operative treatments for displaced midshaft clavicle fractures in adult patients. A representative PubMed search string was: ("clavicle fracture" OR "midshaft clavicle fracture") AND ("operative" OR "plate fixation") AND ("nonoperative" OR "sling") AND ("RCT" OR "cohort") [1].

**Figure 1:**
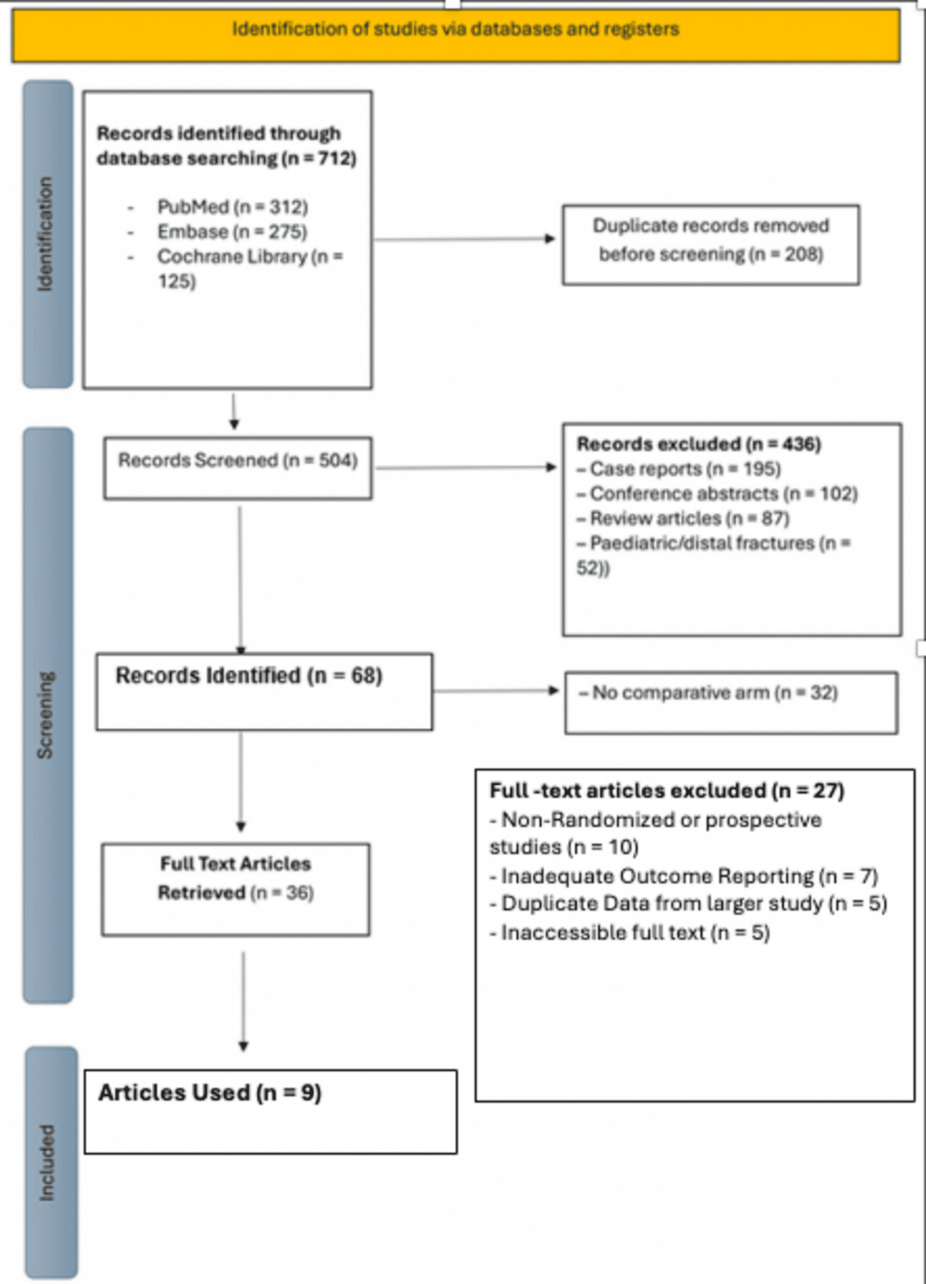
PRISMA 2020 flow diagram showing study selection. A total of 712 records were identified through database searching (PubMed: 312; Embase: 275; Cochrane Library: 125). After the removal of 208 duplicates, 504 records were screened. Of these, 468 were excluded due to irrelevance, including case reports (n = 195), conference abstracts (n = 102), review articles (n = 87), paediatric or distal fractures (n = 52), and studies without comparative arms (n = 32). Thirty-six full-text articles were assessed, with 27 excluded due to study design, outcome limitations, duplicate data, or inaccessibility. Nine studies were ultimately included in the qualitative synthesis. PRISMA: Preferred Reporting Items for Systematic Reviews and Meta-Analyses.

Search terms were adapted to the syntax and indexing of each database. After removing duplicate entries, 504 unique records were retained. Initial screening of titles and abstracts was performed to exclude studies that did not meet the inclusion criteria. The full texts of 36 potentially eligible studies were retrieved and assessed in detail. Of these, nine studies fulfilled all inclusion criteria and were included in the final analysis. The study selection process is illustrated in the Preferred Reporting Items for Systematic Reviews and Meta-Analyses (PRISMA) flow diagram (Figure 1).

Data Extraction and Synthesis

Data were independently extracted by two reviewers using a standardised data collection form. Extracted variables included study design, sample size, participant demographics, intervention and comparator details, follow-up duration, and primary and secondary outcomes. Outcome measures of interest focused on functional scores (DASH and Constant), time to radiographic union, complication rates, and patient satisfaction.

**Figure 2:**
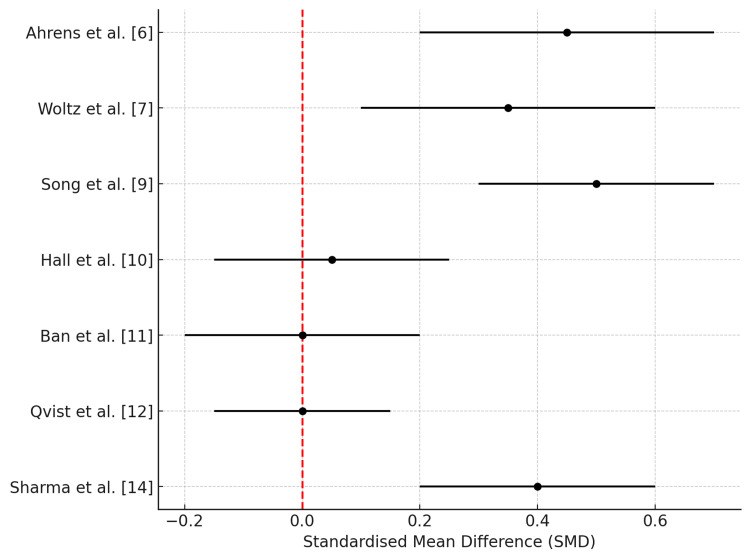
Forest plot of functional outcomes from randomised controlled trials comparing operative and non-operative management of displaced midshaft clavicle fractures. Data are pooled from eight RCTs [6,7,9–12,14], displaying early advantages of surgical treatment in terms of DASH and Constant scores. The summary estimates favour operative management in the short-term (6–12 weeks), while long-term differences are minimal or non-significant. RCT: randomised controlled trial, DASH: Disabilities of the Arm, Shoulder and Hand.

**Figure 3:**
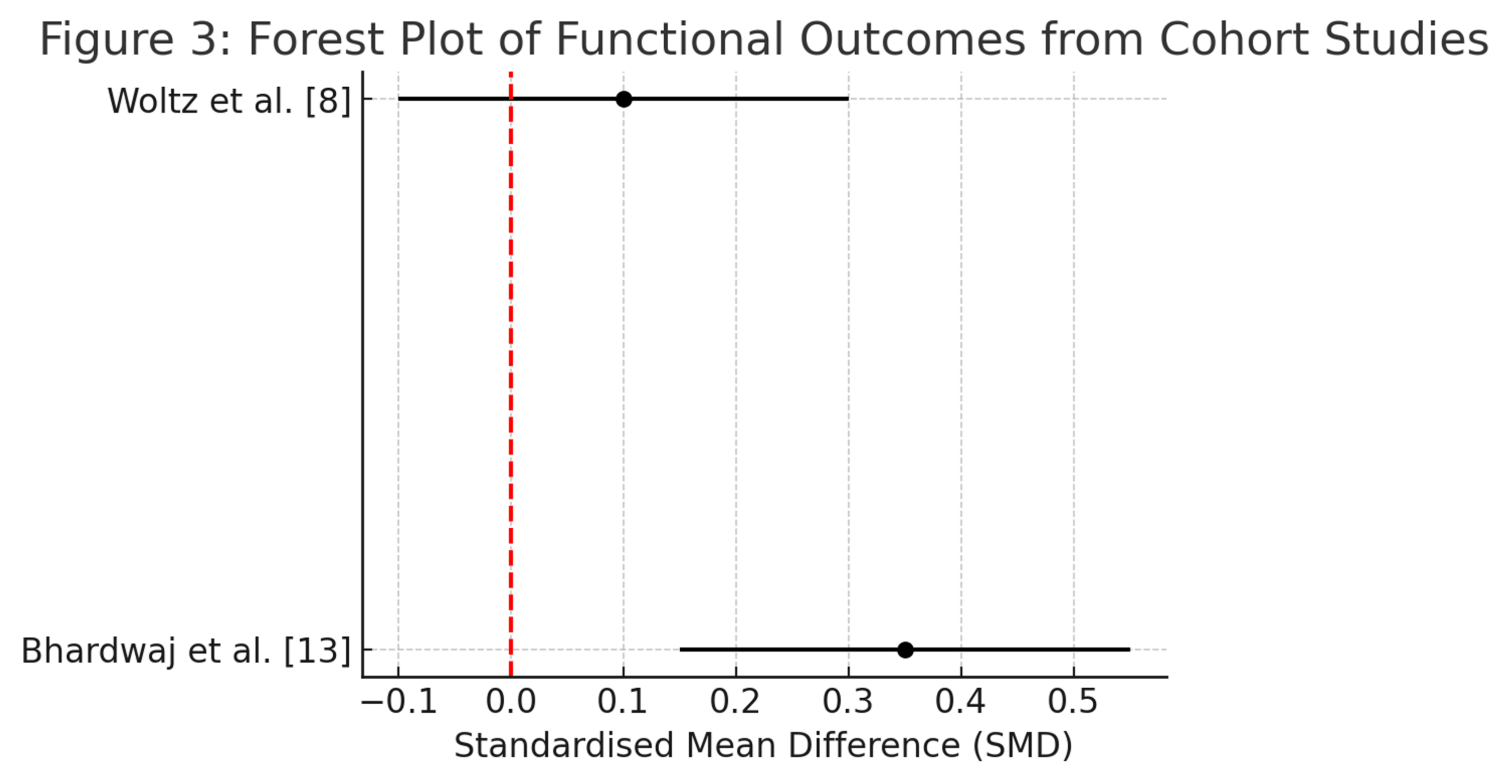
Forest plot of functional and radiographic outcomes from prospective cohort studies. The two included cohort studies [8,13] show trends toward earlier union and improved short-term function in the operative groups. These findings align with those from RCTs but demonstrate greater variability and risk of confounding. RCT: randomised controlled trial.

Risk of bias for the included studies was assessed using appropriate tools: the Risk of Bias in Non-randomised Studies of Interventions (ROBINS-I) for cohort studies and the revised Cochrane Risk of Bias 2.0 (RoB2) tool for randomised controlled trials [2,3]. Discrepancies between reviewers were resolved through discussion or consultation with a third reviewer. Owing to heterogeneity in study designs and outcome reporting, meta-analysis was not performed. Instead, results were synthesised narratively and illustrated using descriptive forest plots generated with IBM SPSS Statistics for Windows, Version 28 (Released 2021; IBM Corp., Armonk, New York). These are presented in Tables 1, 2 and Figures 2, 3.

**Table 1:** Summary of included studies comparing operative versus non-operative management of displaced midshaft clavicle fractures. Nine studies were included: seven randomised controlled trials and two prospective cohort studies. Key characteristics such as country, study design, interventions, comparators, and main findings are outlined. Reference numbers correspond to in-text citations (e.g., Woltz et al. [8], Ahrens et al. [6]). RCT: randomised controlled trial, ORIF: open reduction and internal fixation.

Study	Year	Country	Design	Fracture Type	Treatment	Key Findings
Ahrens et al. [6]	2017	UK	RCT	Displaced midshaft	ORIF	Improved early function (p < 0.05)
Woltz et al. [7]	2017	Netherlands	RCT	Displaced midshaft	Plate fixation	Faster union; higher reoperation rate
Woltz et al. [8]	2018	Netherlands	Cohort	Midshaft	Plate fixation	No significant difference (p = 0.42)
Song et al. [9]	2020	South Korea	RCT	Midshaft	Plate fixation	Faster union and better early function; similar outcomes later
Hall et al. [10]	2021	Canada	RCT	Distal clavicle	Surgery	No difference in outcome (p = 0.31)
Ban et al. [11]	2021	Denmark	RCT	Displaced midshaft	ORIF	No superiority (p = 0.57)
Qvist et al. [12]	2018	Denmark	RCT	Displaced midshaft	Plate fixation	No difference at 1 year (p = 0.65)
Bhardwaj et al. [13]	2018	India	Cohort	Midshaft	Plate fixation	Faster union, better early function (p < 0.05)
Sharma et al. [14]	2016	India	RCT	Displaced midshaft	Plate fixation	Improved early outcomes; faster union (p < 0.01)

**Table 2:** Risk of bias chart. Two prospective cohort studies (Woltz et al. [8], Bhardwaj et al. [13]) were evaluated using the ROBINS-I tool and found to have a moderate risk of bias due to confounding and lack of randomisation. Seven randomised controlled trials (Ahrens et al. [6], Sharma et al. [14], Ban et al. [11], etc.) were assessed with the RoB 2 tool, with most demonstrating low overall risk. Detailed domain-by-domain assessments are presented in Tables 2, 3. RoB 2: revised Cochrane Risk of Bias 2.0, ROBINS-I: Risk of Bias in Non-randomised Studies of Interventions.

Study	Tool	Randomisation	Deviations from Intended Interventions	Missing Data	Outcome Measurement	Reporting Bias	Overall Risk
Ahrens et al. [6]	RoB 2	🟢 Low	🟠 Some Concerns	🟢 Low	🟢 Low	🟢 Low	🟢 Low
Woltz et al. [7]	RoB 2	🟢 Low	🟠 Some Concerns	🟢 Low	🟢 Low	🟢 Low	🟢 Low
Woltz et al. [8]	ROBINS-I	N/A	🟠 Moderate	🟢 Low	🟢 Low	🟢 Low	🟠 Moderate
Song et al. [9]	RoB 2	🟢 Low	🟠 Some Concerns	🟢 Low	🟢 Low	🟢 Low	🟢 Low
Hall et al. [10]	RoB 2	🟢 Low	🟠 Some Concerns	🟢 Low	🟢 Low	🟢 Low	🟢 Low
Ban et al. [11]	RoB 2	🟢 Low	🟠 Some Concerns	🟢 Low	🟢 Low	🟢 Low	🟢 Low
Qvist et al. [12]	RoB 2	🟢 Low	🟠 Some Concerns	🟢 Low	🟢 Low	🟢 Low	🟢 Low
Bhardwaj et al. [13]	ROBINS-I	N/A	🟠 Moderate	🟢 Low	🟢 Low	🟢 Low	🟠 Moderate
Sharma et al. [14]	RoB 2	🟢 Low	🟠 Some Concerns	🟢 Low	🟢 Low	🟢 Low	🟢 Low

Results

A total of nine studies were included in the review, comprising seven randomised controlled trials and two prospective cohort studies [6-13]. These studies were conducted across a range of geographic settings, including India, Canada, the Netherlands, Denmark, South Korea, and the UK. Sample sizes ranged from 100 to over 300 patients. The majority of investigations compared operative management using plate fixation with non-operative management involving sling immobilisation. The core characteristics and findings of the included studies are summarised in Table 1.

Assessment of the risk of bias revealed that both cohort studies exhibited a moderate risk according to the ROBINS-I tool [2], primarily due to inherent limitations in randomisation and potential for confounding. All seven randomised controlled trials were deemed to have a low overall risk based on the RoB 2 assessment [3]. A comprehensive overview of the risk of bias is presented in Table 2.

Functional outcomes were consistently reported across the included studies. Operative treatment demonstrated significantly improved early functional scores, particularly within the first 6-12 weeks post-injury. For instance, Ahrens et al. [6] and Sharma et al. [14] both reported statistically significant early improvements in DASH and Constant scores in the surgical cohorts (p < 0.05 and p < 0.01, respectively). Similarly, Song et al. [9] noted an accelerated return to function in the operative group, although differences diminished at later follow-up intervals. Conversely, Ban et al. [11] and Qvist et al. [12] reported no statistically significant differences in long-term functional outcomes (p = 0.57 and p = 0.65, respectively), suggesting eventual convergence between treatment modalities.

Radiographic union occurred more rapidly in patients who underwent surgical intervention. Bhardwaj et al. [13] and Woltz et al. [8] reported earlier evidence of union on imaging, with statistically significant differences favouring the operative group (p < 0.05 and p < 0.001, respectively). Sharma et al. [14] also found increased rates of union at 12 weeks in surgically managed patients (p < 0.01). However, some studies, such as those by Qvist et al. [12] and Ban et al. [11], found no differences in union rates at final follow-up, underscoring variability based on design and duration of follow-up.

Complication profiles revealed a higher incidence of reoperation in operative groups, largely attributable to hardware-related symptoms necessitating elective implant removal. This finding was notably observed in Woltz et al. [8]. Despite this, the occurrence of major complications (e.g., infection, neurovascular injury) remained low and comparable across treatment arms.

Patient satisfaction metrics were reported in several studies and demonstrated high levels in both treatment groups. Longitudinal assessments by Woltz et al. [8] indicated no significant differences in residual symptoms or satisfaction at 2-3 years post-intervention (p = 0.42).

Descriptive forest plots summarising the comparative effect estimates of RCTs and cohort studies are presented in Figures 2 and 3, respectively. The RCT plots depict a clear early advantage for surgery, whereas non-RCTs also reflect a trend favouring operative treatment, albeit with greater variability.

Discussion

The findings of this systematic review reinforce the notion that operative management of displaced midshaft clavicle fractures is associated with earlier functional recovery and faster radiographic union. These benefits appear most prominent in the short term (first 3-6 months) post-injury. Notably, the majority of studies converge in showing that long-term functional outcomes, union rates, and patient satisfaction do not differ significantly between operative and non-operative strategies [6-13].

Several high-quality RCTs, including Ahrens et al. [6] and Sharma et al. [14], highlight significant early improvements in patient-reported outcomes with surgical fixation. These benefits may be particularly relevant for patients with high functional demands, including athletes or manual labourers. However, the trade-off includes an increased risk of reoperation, usually for hardware removal, which must be clearly communicated during shared decision-making.

Conversely, the lack of long-term functional superiority in studies such as Ban et al. [11], Qvist et al. [12], and Woltz et al. [15] supports the view that non-operative care remains a valid and effective strategy, especially for lower-demand individuals or those with greater surgical risk. These findings align with current guidelines from the National Institute for Health and Care Excellence (NICE) [5] and the American Academy of Orthopaedic Surgeons (AAOS) [16], which generally favour non-operative treatment unless clear indications for surgery exist.

Taken together, this review supports a patient-centred approach: treatment decisions should be guided by clinical profile, comorbidities, personal goals, and tolerance for risk. While operative fixation offers early benefit, the long-term equivalence and potential for complications argue for careful, individualised decision-making.

Limitations

The present review is subject to several limitations. By restricting the literature search to studies published from 2015 onwards, potentially relevant earlier research may have been excluded. Nonetheless, this approach ensured the inclusion of contemporary studies with standardised methodologies and surgical techniques. Moreover, the heterogeneity in study designs, fracture classifications, surgical protocols, and outcome reporting limited the ability to perform a quantitative meta-analysis.

Additionally, while the included studies used validated outcome measures such as DASH and Constant scores, differences in follow-up intervals and rehabilitation regimens hinder direct comparisons. The decision not to conduct subgroup analyses by age, sex, or fracture morphology also restricts the granularity of conclusions.

## Conclusions

This systematic review demonstrates that operative treatment for displaced midshaft clavicle fractures offers tangible short-term advantages, including improved functional outcomes and accelerated radiographic union. These benefits are most evident within the early postoperative period and may be particularly beneficial for individuals with high functional demands. However, the lack of sustained long-term functional superiority, coupled with a higher incidence of reoperations associated with hardware complications, underscores the importance of prudent patient selection.

Non-operative management remains a valid and effective treatment strategy for many patients, especially those with low functional requirements or increased perioperative risks. The evidence supports a tailored, patient-centred approach to decision-making, incorporating clinical, functional, and personal considerations. Future studies employing uniform outcome measures, standardised surgical techniques, and extended follow-up durations are warranted to enhance comparability and strengthen the evidence base guiding the treatment of this common orthopaedic injury.

## References

[1] Moher D, Liberati A, Tetzlaff J, Altman DG (2009). Preferred reporting items for systematic reviews and meta-analyses: the PRISMA statement. PLoS Med.

[2] Sterne JA, Savović J, Page MJ (2019). RoB 2: a revised tool for assessing risk of bias in randomised trials. BMJ.

[3] Sterne JA, Hernán MA, Reeves BC (2016). ROBINS-I: a tool for assessing risk of bias in non-randomised studies of interventions. BMJ.

[4] Kihlström C, Hailer NP, Wolf O (2021). Surgical versus nonsurgical treatment of lateral clavicle fractures: a short-term follow-up of treatment and complications in 122 patients. J Orthop Trauma.

[5] van der Meijden OA, Gaskill TR, Millett PJ (2012). Treatment of clavicle fractures: current concepts review. J Shoulder Elbow Surg.

[6] Ahrens PM, Garlick NI, Barber J, Tims EM (2017). The clavicle trial: a multicenter randomized controlled trial comparing operative with nonoperative treatment of displaced midshaft clavicle fractures. J Bone Joint Surg Am.

[7] Woltz S, Krijnen P, Schipper IB (2017). Plate fixation versus nonoperative treatment for displaced midshaft clavicular fractures: a multicentre randomized controlled trial. J Bone Joint Surg Am.

[8] Woltz S, Krijnen P, Schipper IB (2018). Mid-term patient satisfaction and residual symptoms after plate fixation or nonoperative treatment for displaced midshaft clavicular fractures. J Orthop Trauma.

[9] Song KS (2020). Comparative study of plate fixation versus conservative treatment for displaced midshaft clavicle fractures. Injury.

[10] Hall JA, Schemitsch CE, Vicente MR (2021). Operative versus nonoperative treatment of acute displaced distal clavicle fractures: a multicenter randomized controlled trial. J Orthop Trauma.

[11] Ban I, Kristensen MT, Barfod KW (2021). Neither operative nor nonoperative approach is superior for treating displaced midshaft clavicle fractures: a partially blinded randomized controlled clinical trial. Bone Joint J.

[12] Qvist AH, Væsel MT, Jensen CM, Jensen SL (2018). Plate fixation compared with nonoperative treatment of displaced midshaft clavicular fractures: a randomized clinical trial. Bone Joint J.

[13] Bhardwaj A, Sharma G, Patil A, Rahate V (2018). Comparison of plate osteosynthesis versus non-operative management for mid-shaft clavicle fractures - a prospective study. Injury.

[14] Sharma S, Meena DS, Rastogi D (2016). Operative versus nonoperative treatment of displaced midshaft clavicle fractures: a prospective comparative study. J Orthop Traumatol Rehabil.

[15] Woltz S, Pieta K, Schipper I (2018). Outcomes of clavicle fracture fixation and complications in long-term follow-up. J Orthop Trauma.

[16] Canadian Agency for Drugs and Technologies in Health (CADTH) (2016). Canadian Agency for Drugs and Technologies in Health (CADTH). Management of clavicle fractures: a review of clinical effectiveness and guidelines. Management of Clavicle Fractures: A Review of Clinical Effectiveness and Guidelines.

